# Which nets are being used: factors associated with mosquito net use in Amhara, Oromia and Southern Nations, Nationalities and Peoples' Regions of Ethiopia

**DOI:** 10.1186/1475-2875-10-92

**Published:** 2011-04-17

**Authors:** Jeremiah M Ngondi, Patricia M Graves, Teshome Gebre, Aryc W Mosher, Estifanos B Shargie, Paul M Emerson, Frank O Richards

**Affiliations:** 1 The Carter Center, Atlanta, GA, USA; 2 Universisty of Cambridge, Cambridge, UK; 3 The Carter Center, Addis Ababa, Ethiopia; 4 Strategic Information Team, The Global Fund to Fight AIDS, Tuberculosis and Malaria, Chemin de Blandonnet 8, Geneva 1214 Vernier, Switzerland

## Abstract

**Background:**

There has been recent large scale-up of malaria control interventions in Ethiopia where transmission is unstable. While household ownership of long-lasting insecticidal nets (LLIN) has increased greatly, there are concerns about inadequate net use. This study aimed to investigate factors associated with net use at two time points, before and after mass distribution of nets.

**Methods:**

Two cross sectional surveys were carried out in 2006 and 2007 in Amhara, Oromia and SNNP regions. The latter was a sub-sample of the national Malaria Indicator Survey (MIS 3R). Each survey wave used multi-stage cluster random sampling with 25 households per cluster (224 clusters with 5,730 households in Baseline 2006 and 245 clusters with 5,910 households in MIS 3R 2007). Net ownership was assessed by visual inspection while net utilization was reported as use of the net the previous night. This net level analysis was restricted to households owning at least one net of any type. Logistic regression models of association between net use and explanatory variables including net type, age, condition, cost and other household characteristics were undertaken using generalized linear latent and mixed models (GLLAMM).

**Results:**

A total of 3,784 nets in 2,430 households were included in the baseline 2006 analysis while the MIS 3R 2007 analysis comprised 5,413 nets in 3,328 households. The proportion of nets used the previous night decreased from 85.1% to 56.0% between baseline 2006 and MIS 3R 2007, respectively. Factors independently associated with increased proportion of nets used were: LLIN net type (at baseline 2006); indoor residual spraying (at MIS 3R 2007); and increasing wealth index at both surveys. At both baseline 2006 and MIS 3R 2007, reduced proportion of nets used was independently associated with increasing net age, increasing damage of nets, increasing household net density, and increasing altitude (>2,000 m).

**Conclusion:**

This study identified modifiable factors affecting use of nets that were consistent across both surveys. While net replacement remains important, the findings suggest that: more education about use and care of nets; making nets more resistant to damage; and encouraging net mending are likely to maximize the huge investment in scale up of net ownership by ensuring they are used. Without this step, the widespread benefits of LLIN cannot be realized.

## Background

Malaria is a leading cause of morbidity and mortality in Ethiopia, being the most frequent cause of outpatient visits and admissions in 2008[1]. Ethiopia was one of the first countries in sub-Saharan Africa to embrace the concept of scaling up for impact (SUFI) in its 2006-2010 national five-year strategic plan for malaria prevention and control, which committed to 100% coverage with, on average, two long-lasting insecticidal nets (LLIN) per household in malarious areas, and 100% access to effective and affordable malaria treatment[2]. The distribution of about 20 million nets since 2006 has meant that Ethiopia has moved from being one of the African countries with the lowest insecticide treated net (ITN) ownership to one of the highest[3].

A Demographic and Health Survey (DHS) in 2005 [4] and a national Malaria Indicator Survey (MIS) in 2007 [5] have been carried out in Ethiopia. A large representative household survey in malarious areas of the three largest regions was also done in 2006[6,7]. These surveys all assessed ITN ownership and use, with the 2006 and 2007 surveys also assessing these indicators specifically for LLIN. The results showed that the proportion of households owning at least one ITN increased from 6.5% of households in 2005[4] to 19.6% (LLIN) in 2006 [6,7] and 65.6% (LLIN) in 2007 [5]. The mean ITN per household was less than 0.1 in 2005, 0.3 (LLIN) in 2006 and 1.2 (LLIN) in 2007. In all households, the proportion of children under five using nets increased from 2% in 2005 to 31.8% in 2006 and to 42.5% in 2007, while a similar increase in the percent of pregnant women using nets was observed (1% in 2005, 35.9% in 2006 and 41.0% in 2007)[5]. However, while overall net use increased greatly to quite a high level due to increased ownership, as previously reported [8] within net owning households the proportion of participants who slept under a net decreased between 2006 and 2007: from 70.8% to 50.2% among persons of all ages; from 70.8% to 58.7% among children under five; and from 81.2% to. 66.1% among pregnant women.

Closer examination of the results obtained in the 2006 and 2007 surveys to shed further light on the patterns of net use has been reported by Shargie *et al *[8]. As expected, household net ownership and percentage of individuals using them increased dramatically between the surveys when assessed in all households. However, when the comparison was restricted to only households owning nets, there was a decline in the proportion of individuals using nets in all regions and population groups that could not be explained by climate factors between years or sampling differences between the 2006 and 2007 surveys[8]. This study aimed to investigate both household and net factors associated with the likelihood of nets being used at two time points in Ethiopia: the 2006 survey and the MIS 2007 survey subsample from Amhara, Oromia and Southern Nations, Nationalities and Peoples' (SNNP) Regions of Ethiopia.

## Methods

### The study setting and surveys

**Figure 1:**
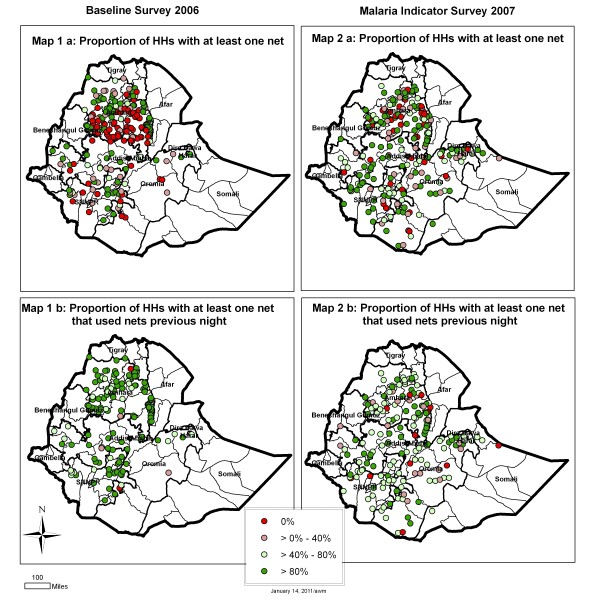
**Map of Amhara, Oromia and SNNP Regional States showing location of clusters in the baseline-2006 (Maps 1a and 1b) and MIS 3R 2007 (Maps 2a and 2b) surveys**. Clusters are color coded by proportion according to the legend. Maps 1a and 2a (net ownership), show the proportions of households in each cluster owning at least one net. Maps 2a and 2b (net use) show **t**he proportions of households in each cluster that were using at least one net the previous night, out of all household owning nets in the cluster. Therefore in Maps 2a and 2b, clusters where no households owned nets are not shown.

This study was conducted in the Amhara, Oromia and Southern Nations, Nationalities and Peoples' (SNNP) regions of Ethiopia (Figure 1, Map). The characteristics, survey design and sampling of for the two surveys are summarized in Table 1. The baseline survey was conducted from December 2006 to January 2007. The sample size estimation and sample selection process have been described previously[6,7]. In brief, a multi-stage cluster random sampling with probability proportional to population size was used to select 224 clusters, and 25 households were randomly selected in each cluster. The sampling frame included only clusters that were defined by the Regional Health Bureaus as malarious (i.e. where more than 10% of the population lived in malarious areas). Clusters were defined as *kebeles *(the smallest administrative unit with an average population of 5,000). Interviews regarding malaria indicators were conducted in the 25 households selected.

**Table 1:** Characteristics of the baseline 2006 and the MIS 3R surveys

Characteristic	Baseline 2006	MIS 3R 2007
Scope of the survey	Surveys in Amhara, Oromia and SNNP regions only	This is a sub-sample of the National MIS comprising 3 regions (Amhara, Oromia and SNNPR).
Dates of survey	December 2006 to January 2007	October through December 2007
Sampling frame	Sampling frame included only *kebeles *defined by the Regional Health Bureaus as malarious by expert knowledge.	Nationally representative sample stratified by three domains: areas below 1,500 m, rural areas between 1,500 m and 2,500 m, and urban areas between 1,500 m and 2,500 m
Primary Sampling Unit	Defined as *kebeles *(the smallest administrative unit with an average population of 5,000) selected with probability proportional to population size	Defined as census enumeration areas (EA) with each cluster comprising an estimated population of 200 households.
Sampling of clusters	5 State-teams per kebele selected randomly from list of state-teams in the selected kebeles.	EA selected randomly with probability proportional to population size of strata.
Sampling of households	5 households per state team using the random-walk method.	Simple random sample of all households in EA selected after mapping all households using personal digital assistants (PDAs) with global positioning system (GPS) capability
Sample size	224 clusters of 25 households	245 clusters of 25 households

Starting in 2006, almost 20 million LLIN (mostly Permanet^® ^2.0, Vestergaard Frandsen, Switzerland) donated by The Global Fund, UNICEF, The Carter Center and other donors were distributed to all areas deemed by Regional Health Bureaus to be 'malarious' by expert knowledge. The target ownership level was an average of 2 LLIN per household in such areas. The Regions Health Bureaus decided separately on priority areas, methods and household entitlement for distribution. The LLIN were distributed in stand-alone campaigns by health workers and local administrators, with households receiving variable numbers of LLIN (usually 1, 2 or 3) based on the number of persons in the household.

The malaria indicator survey (MIS) was conducted from October through December 2007 and has been described elsewhere[5]. In brief, a two-stage cluster random sampling design was used to select a nationally representative sample stratified by three domains: areas below 1,500 m, rural areas between 1,500 m and 2,500 m, and urban areas between 1,500 m and 2,500 m. Clusters were defined as census enumeration areas (EA) with each cluster comprising an estimated population of 200 households. In Amhara and Oromia regions, there was over-sampling of clusters to generate samples for estimating malaria indicators at region level. The sample size thus allowed for detection of changes in malaria indicators between the baseline 2006 and MIS 2007 surveys in the combined three regions covered by the baseline 2006 survey (Amhara, Oromia and SNNPR). In each selected EA, all households were mapped, and 25 households were randomly selected by use of personal digital assistants (PDAs) with global positioning system (GPS) capability. Interviews regarding malaria indicators were conducted in those 25 households.

In this study, the analysis was not restricted to clusters in MIS 2007 located in 'malarious' areas as defined by expert knowledge, altitude or any other system. The results from all clusters in the 3 regions of Amhara, Oromia and SNNPR were included. This is referred to as the MIS 3R 2007 sub-sample.

### Household questionnaire

The baseline and MIS survey questionnaires were both based on the Malaria Indicator Survey Household Questionnaire, modified for the local conditions to include socioeconomic factors[9]. The questionnaire was translated and conducted in Amharic language and pilot-tested in a non-survey cluster to determine the validity of the pre-coded answers. At baseline, a paper-based questionnaire was used while at MIS 2007, the survey was conducted using PDAs. Interviews were conducted with the head of household, or another adult if the head of household was absent or unable to respond for any reason.

Respondents were asked about: presence, number, type, and condition of mosquito nets (verified by observation for hole size referencing 'torch battery' size D cell, diameter 33 mm, for hole size reference); nets used the previous night; duration since acquisition of nets; and source of the nets (purchased or free of charge through mass distribution). Other factors recorded during the interview included: number of people resident in the household; number of sleeping rooms/spaces; children under five and pregnant women in household; and recent (within the last 12 months) indoor residual spraying. Altitude and location of each household were recorded using the Global Positioning System.

### Statistical methods

**Figure 2:**
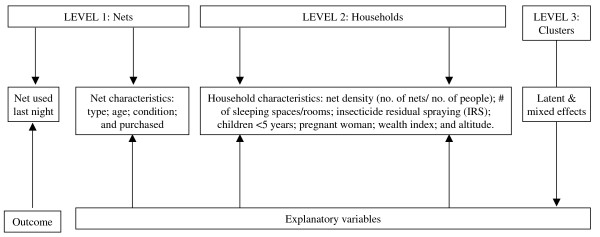
**Summary of data framework for analysis of association between use of net and explanatory factors**.

Figure 2 summarizes the framework for analysis of association between use of net and explanatory factors. This analysis was restricted to households owning nets in each survey. Since this is a net level analysis, it does not take into account the number or ages of the particular individuals under each net. The use (or not) of a particular net the previous night was the dependent variable. Net density was calculated by dividing the number of nets in a household by the number of people in the household to account for the potential correlation between these variables. A quintile household wealth index was derived from relevant household characteristics using principal components analysis as previously described by Graves *et al*[10] and the 20% poorest (lowest quintile) was compared against the 80% richest (quintiles 2-5). Statistical analysis was conducted using Stata 8.2 (Stata Corporation, College Station, Texas). Descriptive statistics were used to examine the characteristics of the sample, and prevalence of outcomes and explanatory factors. To account for differences in the sampling design, prevalence estimates were adjusted for sampling weights. To investigate the association between net use and explanatory factors, hierarchical regression models were developed using generalized linear latent and mixed models (GLLAMM)[11]. The multilevel structure of GLLAMM allowed for non-independence of the household variables, enabled clustering of net observations within households and clusters, and allowed for variability at net, household and cluster levels. Univariate analysis was conducted for each potentially explanatory factor. Multivariable models were then developed by stepwise regression analysis for model selection. This involved starting with a null model then proceeding in a sequential fashion of adding/deleting explanatory variables if they satisfied the entry/removal criterion, which was set at 5% significance level using a log-likelihood ratio test.

### Ethical considerations

The survey protocols received ethical clearance from the Emory University Institutional Review Board (IRB#1816 and 6389), the Centers for Disease Control and Prevention ethical review committee (IRB#990132) and the Ethiopian Science and Technology Agency. For both surveys, informed consent to participate in interviews was sought from the heads of household in accordance with the tenets of the declaration of Helsinki.

## Results

### Characteristics of the sample

**Figure 3:**
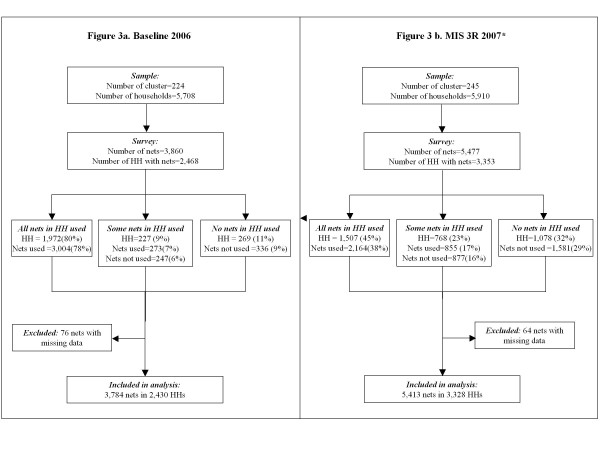
**Flowchart showing the sample population and proportion of nets used the previous night**. HH, households *Amhara, Oromia, and Southern Nations, Nationalities and Peoples' (SNNP) regions

The characteristics of the sample are summarized in Table 2 and Figure 3. A total of 3,784 nets in 2,430 households were included in the baseline 2006 analysis while the MIS 3R 2007 analysis comprised 5,413 nets in 3,328 households. At baseline 2006 survey, 59.4% of the nets were LLIN while in MIS 3R 2007, LLIN comprised 95.1% of the nets. Despite an increase in the proportion of households owning at least one net from 37.0% at baseline 2006 to 56.7% at MIS 3R 2007 (shown visually in Figure 1 by cluster as increase in the proportion of green clusters between panels Map 1a and 2a), a lower proportion of households with nets reported using any nets the previous night during MIS 3R 2007 (shown visually in Figure 1 as a decrease in the proportion of green clusters between Map panels 1b and 2b). The proportion of households with nets in which at least one net was used the previous night was 89.3% at baseline 2006 and 68.1% at MIS 3R 2007. The proportion of all nets reported as used the previous night decreased considerably from 85.1% to 56.0% at baseline 2006 and MIS 3R 2007, respectively (Table 2 and Figure 3).

**Table 2:** Characteristics of sample population

Characteristics	Baseline 2006				MIS 3R 2007*			
		
	Amhara	Oromia	SNNP	Total	Amhara	Oromia	SNNP	Total
Number of clusters	160	32	32	224	108	97	40	245
Number of HHs surveyed	4,101	809	798	5,708	2,609	2,321	980	5,910
Number of participants	19,059	4,428	4,397	27,884	10,733	10,266	4,082	25,081
Number of participants in HHs owning nets	8,298	2,019	2,361	12,678	5,554	1,792	1,080	8,426
								
Proportion of HHs owning nets (%)	40.7	44.4	50.5	42.6	74.4	41.4	46.2	56.7
Proportion of HHs with nets using nets last night (%)	90.3	86.1	87.8	89.3	71.2	60.5	71.0	68.1
								
Number of nets	2,707	526	551	3,784	3,280	1,442	691	5,413
Proportion of nets that were LLIN (%)	54.8	68.8	73.0	59.4	98.5	88.1	99.3	95.8
Proportion of nets used last night (%)	84.0	85.7	89.8	85.1	57.7	50.1	60.3	56.0
Mean number of people per net	2.7	3.1	2.8	2.7	2.4	2.6	2.2	2.4

*Amhara, Oromia, and Southern Nations, Nationalities and Peoples' (SNNP) regions

HHs, households; LLIN, long lasting insecticidal net

### Associations between net use and explanatory factors: baseline survey 2006

Univariate logistic regression analysis of the associations between net use at baseline 2006 and explanatory factors is shown in Table 3. Factors associated with increased proportion of nets used were: LLIN net type (OR = 1.4; 95% CI 1.0-1.8); and increasing wealth index (lowest quintile compared to quintiles 2-5, OR = 1.6; 95% CI 1.2-2.4). Reduced proportion of nets used was associated with: increasing age of the net, from less than 6 months to more than 1 year old (Ptrend < 0.001); unsafe nets (>5 holes) compared to good nets (Ptrend = 0.008); increasing household net density (Ptrend = 0.001); and increasing altitude (>2000 m, OR = 0.2; 95% CI 0.1-0.9).

**Table 3:** Baseline 2006: univariable logistic regression analysis of association of net use and explanatory factors

Factors		Total nets N = 3,784	Nets used last night n = 3,221	Proportion of nets used last night (%)	Odds Ratio	95% CI	*p-value*
*Net characteristics*							
Type of net	Other type	1,536	1,290	84.0	1.0		
	LLIN	2,248	1,931	85.9	1.4	1.0-1.8	0.027
Age of the net	≤6 months	2,847	2,491	87.5	1.0		*Ptrend *<0.001
	>6 months - 1 year	580	481	82.9	0.8	0.5-1.1	
	>1 year	357	249	69.7	0.3	0.2-0.4	
Net condition	Good*	3,430	2,927	85.3	1.0		*Ptrend *= 0.008
	Fair (no holes >33 mm)	174	159	91.4	2.3	1.1-4.7	
	Poor (1-4 holes >33 mm)	95	69	72.6	0.3	0.2-0.6	
	Unsafe (5 or more holes >33 mm)	85	66	77.6	0.5	0.2-1.0	
Net purchased	No	3,654	3,117	85.3	1.0		
	Yes	130	104	80.0	0.6	0.3-1.1	0.101
*Household characteristics*							
Number of sleeping rooms in HH (per additional room)					1.0	0.9-1.3	0.934
Net density^±^	<0.5	2911	2530	86.9	1.0		*Ptrend *<0.001
	≥0.5 < 1.0	762	619	81.2	0.6	0.4-0.8	
	≥1.0 < 1.5	98	67	68.4	0.3	0.1-0.6	
	≥1.5	13	5	38.5	0.03	0.003-0.3	
Insecticide residual spraying	Not sprayed	2,712	2298	84.7	1.0		
	Sprayed within last 12 months	1,072	923	86.1	1.2	0.9-1.7	0.145
Child under 5 years in HH	No	1,379	1,163	84.3	1.0		
	Yes	2,405	2,058	85.6	1.1	0.8-1.4	0.554
Pregnant woman in HH	No	3,441	2,927	85.1	1.0		
	Yes	343	294	85.7	1.1	0.7-1.5	0.695
Wealth index	Lowest quintile (20% poorest)	795	652	82.0	1.0		
	Quintiles 2-5 (80% richest)	2,989	2,569	85.9	1.7	1.2-2.4	0.002
Altitude	<1000 m	64	60	93.8	1.0		
	≥1000 - ≤2000 m	2,986	2552	85.5	0.3	0.8-1.1	0.07
	>2000 m	734	609	83.0	0.2	0.1-0.9	0.035

HH, household; LLIN, long lasting insecticidal net;

*275 nets still in package and unused

^±^number of nets/number of people in household

Table 4 shows the multivariable associations between net use at baseline 2006 and explanatory factors. Factors independently associated with increased proportion of nets used were: LLIN net type (OR = 1.4; 95% CI 1.1-1.8); and increasing wealth index (lowest quintile compared to quintiles 2-5, OR = 1.6; 95% CI 1.2-2.2). Reduced proportion of nets used was independently associated with: increasing net age (Ptrend<0.001); increasing household net density (Ptrend<0.001); poor net condition (OR = 0.5; 95% CI 0.2-0.9); and increasing altitude (>2000 m, OR = 0.1; 95% CI 0.04-0.6).

**Table 4:** Baseline 2006: multivariable logistic regression analysis of association of net use and explanatory factors

Factors		Odds Ratio	95% CI	p-value
Net type (LLIN)		1.4	1.1-1.8	0.018
Age of the net	>6 months - 1 year	0.8	0.5-1.1	*Ptrend *<0.001
	>1 year	0.3	0.2-0.4	
Net condition	Fair (no holes >33 mm)	2.6	1.3-5.2	0.007
	Poor (1-4 holes >33 mm)	0.5	0.2-0.9	0.018
	Unsafe (5 or more holes >33 mm)	0.7	0.3-1.5	0.307
Net density	≥0.5 < 1.0	0.5	0.4-0.7	*Ptrend *<0.001
	≥1.0 < 1.5	0.2	0.1-0.5	
	≥1.5	0.03	0.04-0.2	
Household wealth index: quintiles 2-5 (80% richest)		1.8	1.2-2.2	0.002
Altitude	≥1,000 - ≤2,000 m	0.2	0.1-0.7	0.012
	>2,000 m	0.1	0.04-0.6	0.007

LLIN, long lasting insecticidal net

### Associations between net use and explanatory factors: MIS 3R 2007 survey

Table 5 summarizes the univariate logistic regression analysis of the associations between net use at MIS 2007 and explanatory factors. Factors associated with increased proportion of nets used were: LLIN net type (OR = 1.7; 95% CI 1.2-2.4); indoor residual spraying (OR = 1.5; 95% CI 1.2-1.8); and increasing wealth index (lowest quintile compared to quintiles 2-5, OR = 1.2; 95% CI 1.0-1.4). Reduced proportion of nets used was associated with: increasing age of the net (Ptrend<0.001); unsafe nets compared to good nets (Ptrend<0.001); increasing household net density (net density >1.5, OR = 0.3; 95% CI 0.1-0.8); and increasing altitude (>2000 m, OR = 0.6; 95% CI 0.4-0.8).

**Table 5:** MIS 3R 2007*: univariable logistic regression analysis of association of net use and explanatory factors

Factors		Total nets N = 5,413	Nets used last night n = 3,031	Proportion of nets used last night (%)	Odds Ratio	95% CI	*p-value*
*Net characteristics*							
Type of net	Other type	225	107	47.6	1.0		
	LLIN	5,188	2,924	56.4	1.7	1.2-2.4	0.006
Age of the net	≤6 months	1,888	1,112	58.9	1.0		<0.001
	>6 months - 1 year	2,491	1,465	58.8	1.0	0.8-1.2	
	>1 years	1034	454	43.9	0.5	0.4-0.6	
Net condition	Good**	3,890	2,245	57.7	1.0		*Ptrend *<0.001
	Fair (no holes >33 mm)	767	475	61.9	1.3	1.1-1.6	
	Poor (1-4 holes >33 mm)	485	242	49.9	0.7	0.5-0.9	
	Unsafe (5 or more holes >33 mm)	271	69	25.5	0.2	0.1-0.3	
Net purchased	No	5,161	2,885	55.9	1.0		
Yes	252	146	57.9	1.1	0.8-1.5	0.547	
*Household characteristics*							
Number of sleeping rooms in HH (per additional room)					1.1	0.9-1.2	0.455
Net density^±^	<0.5	3,173	1799	56.7	1.0		
	≥0.5 < 1.0	1,825	1018	55.8	1.0	0.8-1.1	0.57
	≥1.0 < 1.5	379	202	53.3	0.9	0.7-1.2	0.407
	≥1.5	36	12	33.3	0.3	0.1-0.8	0.018
Insecticide residual spraying	Not sprayed	4,317	2356	54.6	1.0		
	Sprayed within last 12 months	1,096	675	61.6	1.5	1.2-1.8	<0.001
Child under 5 years in HH	No	2,423	1,368	56.5	1.0		
	Yes	2,990	1,663	55.6	0.9	0.8-1.1	0.471
Pregnant woman in HH	No	5,041	2,832	56.2	1.0		
	Yes	372	199	53.5	0.9	0.7-1.6	0.336
Wealth index	Lowest quintile (20% poorest)	1,183	631	53.3	1.0		
	Quintiles 2-5 (80% richest)	4,230	2,400	56.7	1.2	1.0-1.4	0.037
Altitude	<1,000 m	4,716	2649	56.2	1.0		
	≥1,000 - ≤2,000 m	514	301	58.6	1.1	0.9-1.6	0.328
	>2,000 m	183	81	44.3	0.6	0.4-0.8	0.005

*Amhara, Oromia, and Southern Nations, Nationalities and Peoples' (SNNP) regions

**373 nets still in package and unused

^±^number of nets/number of people in household

Multivariable associations between net use at MIS 3R 2007 and explanatory factors are shown in Table 6. Factors independently associated with increased proportion of nets used were: indoor residual spraying (OR = 1.5; 95% CI 1.2-1.8); and increasing wealth index (lowest quintile compared to quintiles 2-5, OR = 1.2; 95% CI 1.0-1.5). Reduced proportion of nets used was independently associated with: increasing net age (Ptrend<0.001); deteriorating net condition (Ptrend<0.001); increasing household net density (Ptrend<0.009); and increasing altitude (>2000 m, OR = 0.7; 95% CI 0.04-1.0).

**Table 6:** MIS 3R 2007*: multivariable logistic regression analysis of association of net use and explanatory factors

Factors		Odds Ratio	95% CI	p-value
Age of the net	>6 months - 1 year	1.0	0.9-1.2	*Ptrend *<0.001
	>1 year	0.6	0.5-0.7	
Net condition	Fair (no holes >33 mm)	1.3	1.1-1.7	*Ptrend *<0.001
	Poor (1-4 holes >33 mm)	0.8	0.6-1.0	
	Unsafe (5 or more holes >33 mm)	0.2	0.2-0.3	
Net density	≥0.5 < 1.0	0.9	0.6-1.1	*Ptrend *= 0.009
	≥1.0 < 1.5	0.8	0.6-1.1	
	≥1.5	0.3	0.1-0.8	
House sprayed with insecticide		1.5	1.3-1.8	<0.001
Household wealth index: quintiles 2-5 (80% richest)		1.2	1.0-1.5	0.027
Altitude	≥1,000 - ≤2,000 m	1.2	1.0-1.6	0.102
	>2,000 m	0.7	0.4-1.0	0.036

*Amhara, Oromia, and Southern Nations, Nationalities and Peoples' (SNNP) regions

## Discussion

This paper describes factors associated with net use based on two large cross-sectional malaria indicator surveys in three regions of Ethiopia. Following mass distribution of nets, a large increase in the proportion of households owning LLINs in 2007 compared to 2006 was observed; however, there was an apparent decline in the proportion of nets used between 2006 and 2007[8]. For this reason this investigation of the factors associated with net use at the two surveys using multilevel logistic regression modeling was undertaken. In 2006, LLIN were more likely to be used than non-LLIN; this association had disappeared by 2007 when the great majority of nets owned were LLIN. The association between IRS in households and increased net use at MIS 3R 2007 possibly arises from the fact that IRS is done in higher risk malarious areas, where the population perceives greater risk of malaria.

The study has a number of potential limitations. Firstly, the outcome of whether nets were used was based on self-reporting, and in cross-sectional surveys seasonality may influence reported net use behaviour depending on the perceived risk of malaria. Moreover, reported use of nets 'the previous night' only captures a cross-section of use at one-night in time and thus provides somewhat unclear indication of regular use. Secondly, while both surveys employed multistage cluster sampling, the sampling frames were slightly different. At baseline 2006, only areas defined as "malarious" (program target areas defined by expert knowledge) were included in the sampling frame. In MIS 3R 2007, the sampling frame was all areas below 2500 m, stratified to three domains: areas below 1500 m, rural areas between 1500 m and 2500 m, and urban areas between 1500 m and 2500 m. In 2006 survey, primary sampling units (PSUs) were defined as *kebeles *(the smallest administrative unit with an average of 1000 households) at baseline survey while in MIS 3R 2007, the PSUs were defined as census enumeration areas (with an average of 200 households in each). To partially account for the sampling differences between the two surveys, we used the multilevel sample weights estimated for each survey. Sensitivity analysis comparing the "malarious" (by expert knowledge definition) and non-malarious clusters from MIS 3R 2007 did not reveal any differences in household net ownership and proportion of nets used. Finally, the two surveys were conducted nearly one year apart but at slightly different times, although both followed as soon as possible after the generally accepted main rainy season of July-August, which usually precedes the peak malaria season over the next few months. If anything, the slightly earlier timing of the MIS 2007 would bias towards greater net use. Previously, evidence from climate data sources showed that neither rainfall differences between the survey years nor increase in mean temperature at the time of the surveys were likely to have biased household net use[8].

In general increased availability of nets in households, as assessed in cross sectional surveys, is associated with increased net use[12,13]. While these previous studies investigated factors associated with net use by household participants, this analysis investigated factors affecting use at the net level. The study found that in both surveys, increasing household net density, especially net density of 1.5 or more, meant lower likelihood of use for individual nets, suggesting that there is a degree of saturation of net ownership after the mass scale up. The study also identified a number of additional factors influencing net use at household level both in 2006 and 2007, suggesting that net oversupply within households is not the only reason for the decline in proportion of all nets being used.

There are relatively few countries where nets have been available at scale for a long enough period to examine patterns of net use over an extended period, but it is known that the factors determining net use are complex[14-17]. The knowledge of even how to hang a net correctly, or the materials needed to do so, may be lacking[18]. Factors that have been associated with net use include age, educational level attained, wealth, urban/rural location, seasonality and weather[19-24]. While Ethiopian women's and mothers' knowledge about malaria is positively associated with both their own and their children's use [25], over time the occurrence of net loss, deterioration and/or perceived lack of need or impact on disease may cause attrition in net use. This needs to be counteracted by education on net use and care, and by opportunities for net replacement.

Some of the factors associated with net use at both surveys were not surprising: for example older and more damaged nets were less likely to be used. Since we know that nets rapidly sustain physical damage (but not loss of effective insecticide levels) in Ethiopian conditions [26] this suggests that net use will be increased by the supply of stronger and more durable nets, better availability of replacement nets, and perhaps promotion of net mending. In both surveys, nets in households located at higher altitude and where IRS was not warranted were less likely to be used. The abandonment of damaged nets, reduced use where there is little transmission (no IRS), and reduced use at higher altitude are consistent with a rational decision made at the usefulness of the nets at providing protection from malaria. Net owners may perceive that the net is not offering protection - either through wear or low risk of infection - whether or not this perception is actually valid. Nets in households of higher socioeconomic index were more likely to be used in both surveys. Since SES is associated with education, it is likely that better knowledge is involved in this factor, as was observed by Hwang *et al *2010[25].

Overall our results are contrary to the rising trend of net use in Africa, as documented recently in several papers by Noor [21], Noor *et al *[27], and Hanson *et al *[23]. It may be that Ethiopia's relatively unusual unstable transmission and the intense spatial and temporal variation in malaria risk (real and perceived) modifies the relationship between net ownership and use seen in other countries. Better targeting of nets by the malaria control program management units to areas with the highest actual current incidence of malaria cases (irrespective of altitude or climate based predictions of where malaria is or could be) would cover those at highest risk. Nevertheless we believe that longitudinal representative studies such as this one illustrate the possible lack of sustainability of high levels of net use once the novelty or perceived value as a protection from malaria has worn off.

## Conclusions

Although household net ownership increased following mass distribution of LLIN fewer households with nets reported using nets the previous night during MIS 3R 2007 compared to the baseline 2006 survey. The factors affecting net use are more complex than just the level of household net ownership. Following mass distribution of LLIN it was found that a lower proportion of households with nets reported using those nets the previous night compared to baseline. This study has identified modifiable factors affecting net use that could help maximize the huge investment in scale up of net ownership. While net replacement remains important, the findings suggest that: more education about use and care of nets; understanding why nets become damaged so quickly; making nets more resistant to damage; and encouraging net mending are likely to increase use of nets by households. However, further research into net usage after mass distribution campaigns is urgently needed.

## Competing interests

The authors declare that they have no competing interests.

## Authors' contributions

The Ethiopia MIS Working Group undertook the MIS survey; JMN undertook data analysis; JMN & PMG drafted the manuscript which all authors edited and approved.

## Note

The Ethiopia Malaria Indicator Survey Working Group is comprised by: Mekonnen Amena, Laurent Bergeron, Hana Bilak, Brian Chirwa, Firew Demeke, Wubishet Dinkessa, Yeshewamebrat Ejigsemahu, Paul M Emerson, Tekola Endeshaw, Kebede Etana, Gashu Fente, Scott Filler, Anatoly Frolov, Khoti Gausi, Teshome Gebre, Tedros Adhanom Gebreyesus, Alemayehu Getachew, Asefaw Getachew, Patricia M Graves, Zelalem HaileGiorgis, Afework Hailemariam, Jimee Hwang, Daddi Jima, Henok Kebede, Abraham Lilay, Christopher Lungu, Ambachew Medhin, Addis Mekasha, John Miller, Aryc W Mosher, Hussein Muhamed, Sirgut Mulatu, Rory Nefdt, Jeremiah Ngondi, Dereje Olana, Richard Reithinger, Frank O Richards Jr, Amir Seid, Estifanos Biru Shargie, Richard Steketee, Zerihun Tadesse, Tesfaye Teferri, Agonafer Tekalegne, Eskindir Tenaw, Abate Tilahun, Adam Wolkon, Biratu Yigezu, Gedeon Yohannes.
